# Epithelioid Hemangioendothelioma of the Mastoid: Resection for Recurrence and Adjuvant Radiation with 8-Year Followup

**DOI:** 10.1155/2013/469201

**Published:** 2013-01-27

**Authors:** Doniel Drazin, Ravi Gandhi, Elzbieta Slodkowska, Alan S. Boulos

**Affiliations:** ^1^Department of Neurosurgery, Cedars-Sinai Medical Center, Los Angeles, CA 90048, USA; ^2^Division of Neurosurgery, The Neurosciences Institute, Albany Medical Center, Albany, NY 12208, USA; ^3^Department of Pathology, Albany Medical Center, Albany, NY 12208, USA

## Abstract

Epithelioid hemangioendothelioma (EH) is a rare, vascular neoplasm that can affect any age group and has been reported previously in sites including bone, liver, lung, breast, and brain. We describe a case of EH located in the mastoid, which appears to be the first report of EH in this site. The patient was 62 years old when they presented with dizziness and nausea. A suboccipital surgical approach was utilized to resect the tumor. After 18-month followup, the patient was symptom-free; however, imaging demonstrated a recurrence and the patient was taken back to the operating room for a resection. There is no evidence of recurrence after 8 years of followup. This paper aims to reinforce the need for a timely radical excision and aggressive clinical followup as the best hope for cure. Here, we describe an illustrative case and review the pertinent literature.

## 1. Introduction

Weiss and Enzinger first defined a unique soft tissue vascular tumor as an epithelioid hemangioendothelioma (EH) in 1982 [1]. Subsequently, there have been hundreds of cases reported in the literature at sites including the liver, bone, lung, and brain [2–5]. These tumors are rare, representing less than 1% of all vascular tumors but can affect any age group [6]. Intracranial EH has been reported in the parenchyma, along the cavernous sinus, infundibulum, clivus and at the cerebellopontine angle [7–10]. We report the rare case of an epithelioid hemangioendothelioma invading the mastoid bone and adjacent soft tissues in a 62-year-old man.

## 2. Illustrative Case

### 2.1. History

A 62-year-old patient presented with a one-month history of progressive dizziness and nausea. The patient complained of persistent positional vertigo and increasing hearing loss in the left ear. The past medical history was nonsignificant. Neurological examination revealed mild auricular tenderness and decreased hearing, however; the rest of the exam was unremarkable.

### 2.2. Radiographic Evaluation

**Figure 1:**
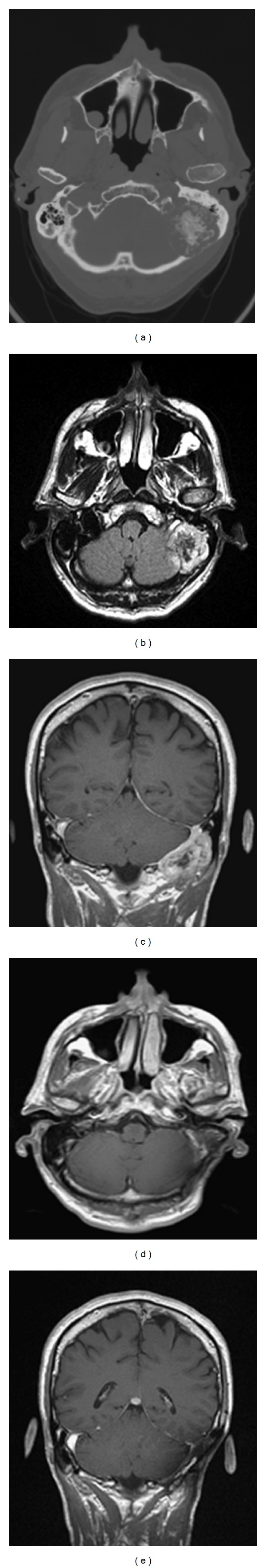
(a) Unenhanced computed tomography scan with bony windowing demonstrates an osteolytic expansile mass within the left mastoid. (b) Gadolinium enhanced axial T1 weighted MRI demonstrates a heterogeneously enhancing lesion within the left mastoid region with mass effect on the cerebellar hemisphere. (c) Gadolinium enhanced coronal T1 weighted MRI demonstrates an enhancing lesion within the left mastoid region extending to the occipital condyle. Postoperative gadolinium enhanced T1 (d) axial and (e) coronal MRI demonstrates a complete resection of the previously identified mastoid mass.

**Figure 2:**
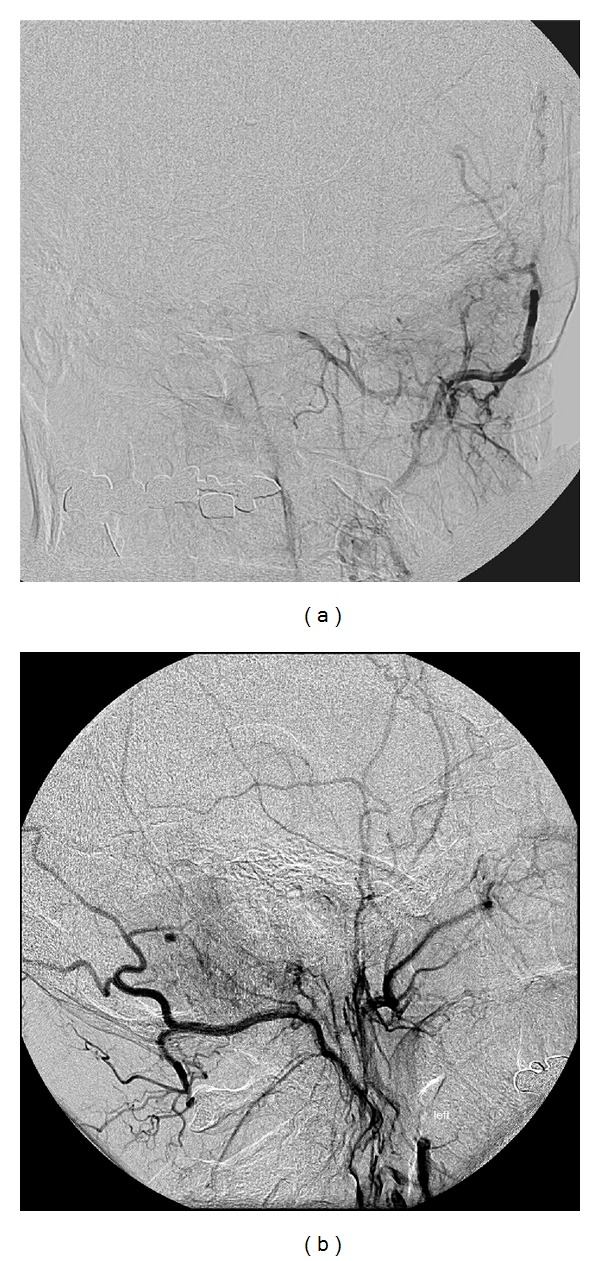
Digitally subtracted angiography (a) AP and (b) lateral projections demonstrating a slight tumor blush from an external carotid injection.

Bone windows on computed tomography (CT) demonstrated an osteolytic mass expanding into the epidural space (Figure 1(a)). The mass extended from the mastoid air cell complex to the occipital condyle. The vestibular aqueduct was in proximity but not affected by the mass. Magnetic resonance imaging (MRI) demonstrated the mass to be hyperintense on T1 weighted with cystic changes and heterogeneously enhancing with gadolinium, and measured 4.5 cm by 3.0 cm by 5.0 cm (Figures 1(b) and 1(c)). The mass compressed the cerebellar hemisphere creating mild mass effect upon the fourth ventricle. Cerebral angiography demonstrated minimal tumor blush supplied by the ascending pharyngeal artery and some from the occipital artery (Figures 2(a) and 2(b)). Embolization of the small feeding vessels was not performed because the feeding vessels were small and would not have a significant impact for the subsequent surgery.

### 2.3. Surgery

The patient underwent tumor resection through a left retromastoid approach utilizing motor evoked potentials, cranial nerve monitoring, and neuronavigation. After careful dissection into the suboccipital triangle, a suboccipital craniectomy extending into the occipital condyle was performed along the dural margin. Special precautions were taken to avoid injuring the underlying dura. The tumor was moderately vascular, but soft, and was resected carefully off the dura and surrounding neural elements. Once we identified the sigmoid sinus, it was carefully skeletonized and we proceeded to the presigmoid region. The VII cranial nerve was identified and skeletonized. We skeletonized the jugular foramen and identified the IXth, Xth, and XIth nerves. The involved mastoid air cells were resected. The semicircular canals were visualized and were uninvolved with tumor. Multiple specimens were sent for frozen section as well as permanent. Once all visible tumor was resected and hemostasis was obtained, an abdominal fat graft was placed and the incision was closed primarily. The patient tolerated the procedure well without any complications.

### 2.4. Histopathological, Immunohistochemical, and Molecular Study

**Figure 3:**
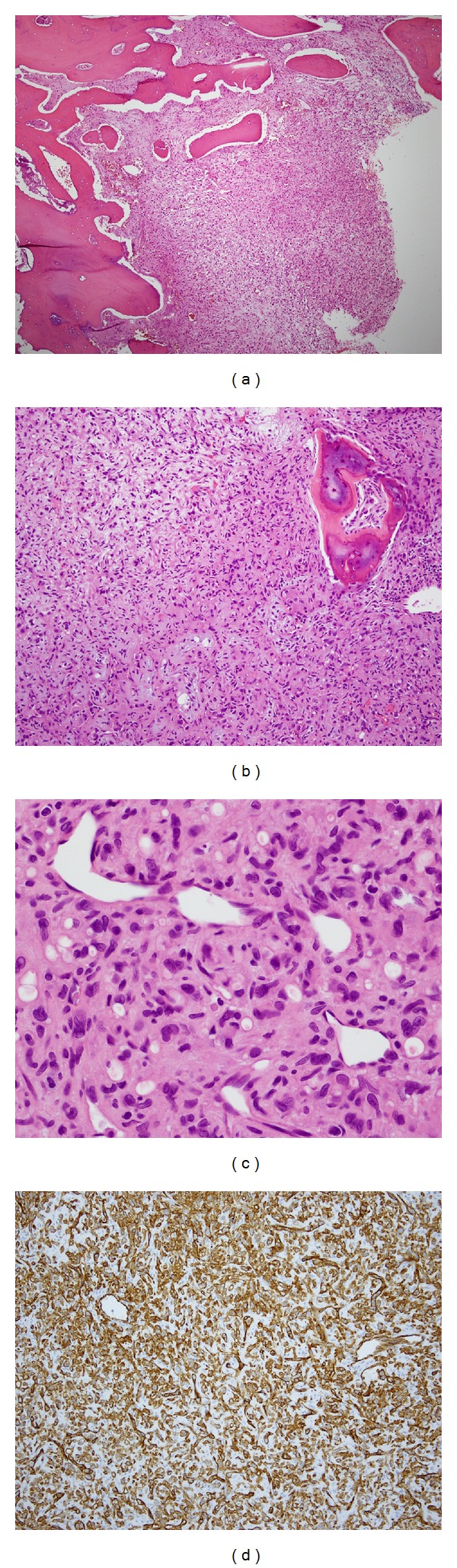
Epithelioid hemangioendothelioma involving the mastoid bone ((a) H&E ×40), composed of cords and sheets of epitheloid cells in the background of fibromyxoid stroma ((b) H&E ×100). Neoplastic cells form cytoplasmic vacuoles ((c) H&E ×200) and strongly and diffusely express endothelial markers ((d) CD31 ×100).

Both specimens showed similar histopathology. There were cords and sheets of epitheloid cells in the background of fibromyxoid stroma (Figure 3(b)). The cells had abundant eosinophilic cytoplasm with cytoplasmic vacuoles representing primitive lumina formation (blister cells), with red blood cells present in some of them (Figure 3(c)). The nuclei were round to elongated, with moderate hyperchromasia and pleomorphism; however, no mitotic activity was present. The neoplastic process involved the mastoid bone (Figure 3(a)) and adjacent soft tissues. Immunohistochemical analysis showed diffuse positivity for endothelial markers, CD31 and CD34 (Figure 3(d)). The neoplastic cells were negative for cytokeratin AE1/AE3 and S100, with low staining with proliferation marker Ki67 (approximately 5%). This morphologic and immunophenotypic picture was that of an epithelioid hemangioendothelioma.

### 2.5. Postoperative Course

**Figure 4:**
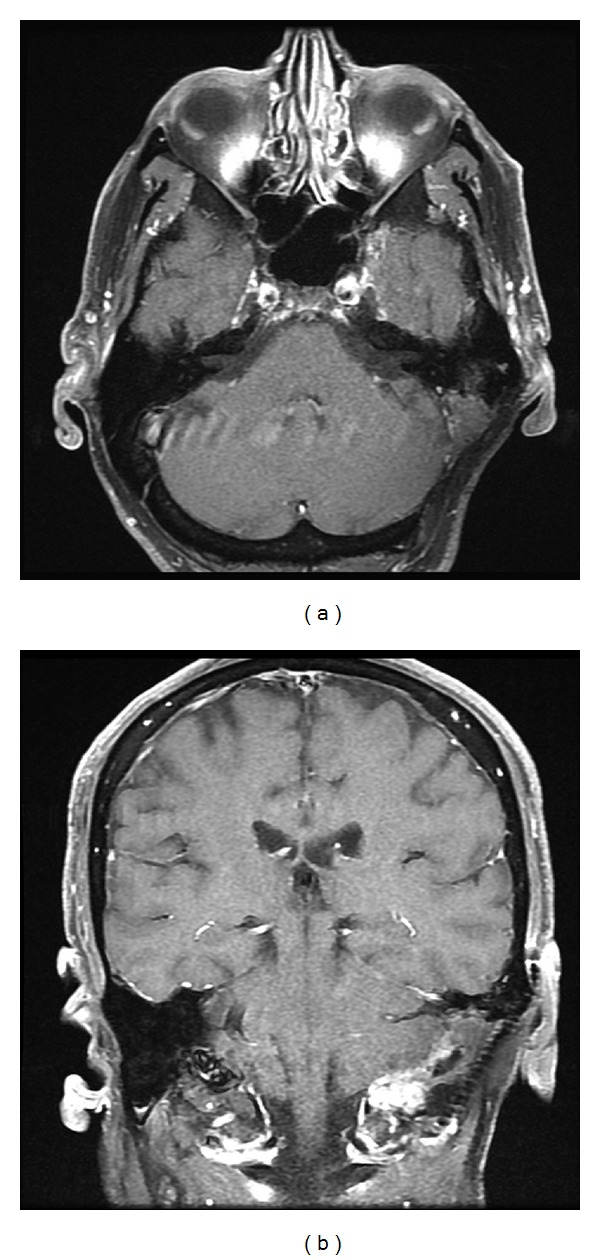
A gadolinium enhanced and fat suppressed T1 (a) axial and (b) coronal demonstrating a recurrent mastoid mass within the previous resection cavity.

Postoperatively, the patient's dizziness resolved with no change in his hearing difficulties or any other cranial nerve deficits. Immediate post-operative imaging demonstrated gross total resection (Figures 1(d) and 1(e)). The patient was referred for radiation therapy; however, based on the resultant gross total resection, careful clinical observation was chosen. MRI done 15 months after resection demonstrated questionable areas of new enhancement; however, the patient wished to continue with observation. A subsequent MRI, however, demonstrated an increase in the size of the area of enhancement (Figures 4(a) and 4(b)). A thallium single-photon emission computed tomography (SPECT) study confirmed the recurrence; therefore, the patient was taken back to the operating room where another surgical excision was performed. Pathological review from the intraoperative specimens confirmed the recurrence of EH. Postoperative imaging demonstrated a persistent region of gadolinium enhancement consistent with residual tumor. The patient was subsequently treated with 33 fractions of radiation, totaling 5940 cGy. Eight years after surgery and radiation, imaging has demonstrated no recurrence and his clinical exam is unchanged.

## 3. Discussion

EH is a rare vascular tumor that has been reclassified as a fully malignant tumor by the World Health Organization [11]. They have been best described in the liver, lung, breast, and bone [2–5]. Intracranial involvement has been described in 36 cases and only 4 other cases of posterior fossa EHs [9, 10, 12, 13]. The risk of malignancy, recurrence, and mestastases and a variable clinical course led to appropriate pathological identification and rigorous follow-up paramount.

The most common reported location is intra-axial with evidence of mass effect causing headache, seizure, or acute neurological deterioration [7]. The variable radiological appearances of these tumors mimic other primary or secondary parenchymal lesions [14]. The CT scan findings range from isodense to hyperdense. The MRI findings are more variable with T1-weighted sequencing ranging from isointense to hyperintense with the most common being heterogeneous [15]. The T2-weighted images are hyperintense or heterogenous. There is usually intense peritumoral vasogenic edema and hetergenous enhancement with gadolinium. The dural-based lesions may mimic meningiomas; however, the cystic conversion and hetergenous enhancement make meningioma less likely [16]. As in our case, the other cases involving the bone were expansile osteolytic masses with intense enhancement [17, 18].

The pathomorphology of EH is very characteristic with cords of epithelioid cells in myxohyaline stroma forming primitive lumina that points towards the endothelial differentiation. The differential diagnosis includes metastatic carcinoma, melanoma, and epithelioid angiosarcoma. The cytoplasmic vacuoles can be confused with gland formation by adenocarcinoma or signet ring cell morphology. However, metastatic neoplasms usually present pronounced nuclear atypia and mitotic activity. Although EH can show some expression of epithelial markers, which is usually focal, adenocarcinoma would be diffusely positive for cytokeratin, as well as mucicarmine [19]. Melanoma, a great mimicker of other tumors, often demonstrates presence of melanin pigment and characteristically strongly expresses S100 protein. Another entity that can look similar is angiosarcoma, especially its epithelioid variant. Some authors believe that there is a continuum between EH and angiosarcoma, with EH being a vascular neoplasm of intermediate malignant potential and angiosarcoma being in the malignant category of vascular tumors [19]. Indeed, angiosarcoma is a more aggressive tumor with a propensity for extensive local growth and metastatic spread to distant organs; it shows a high degree of nuclear atypia and pleomorphism, with high mitotic activity, as opposed to the bland morphology of EH. Also, it has a more primitive morphology of anastomosing slit like spaces, dissecting collagen bundles or sheets of epithelioid cells (which represent very primitive endothelial cells) with the only clue of their true nature being the expression of vascular markers.

About 25% of EH demonstrates “atypical features” of cellular atypia, >1 mitotic figure per 10 hpf, necrosis and spindle morphology; such tumors were historically defined as malignant EH [1]. However, Deyrup et al. [19] in a recent study of 49 EH cases showed that only tumor size and mitotic activity were associated with decreased survival (5-year disease-specific survival of 59%), with tumors with >3 mitotic figures per 50 hpf and size >3.0 cm having the worst prognosis.

The risk of recurrence and metastasis makes treatment complex. Including the current report, there have been six cases of local recurrence, progression, and seeding of the neuraxis [2, 3, 16, 20, 21]. Four of the six cases with progression of disease were reported in cases after a total resection. One of the six cases was given adjuvant radiation and one was given IFN treatment [2, 16]. Complete resection is the treatment of choice when possible and has been associated with a favorable outcome [14]. It is difficult to determine the prognosis based on the histopathology. Parajon and Vaquero [22] reviewed 34 cases of intracranial EH and concluded that if complete surgical resection is achieved, no adjuvant radiotherapy is necessary. However, when presented with clinically aggressive lesions, adjuvant therapy with radiation and Interferon-Alpha has been attempted [17, 20, 22–26]. The rarity of reports makes determining the clinical course of this tumor difficult and therefore, careful clinical followup is warranted.

## 4. Conclusion

The present case describes the clinical and pathologic features of a epithelioid hemangioendothelioma involving the mastoid in a 62-year-old male. To our knowledge, this is the first reported case of EH in this site. Complete surgical resection is the initial treatment of choice, but timely and aggressive clinical followup is necessary to monitor for recurrence.
